# Serious Games in Surgical Medical Education: A Virtual Emergency Department as a Tool for Teaching Clinical Reasoning to Medical Students

**DOI:** 10.2196/13028

**Published:** 2019-03-05

**Authors:** Seung-Hun Chon, Ferdinand Timmermann, Thomas Dratsch, Nikolai Schuelper, Patrick Plum, Felix Berlth, Rabi Raj Datta, Christoph Schramm, Stefan Haneder, Martin Richard Späth, Martin Dübbers, Julia Kleinert, Tobias Raupach, Christiane Bruns, Robert Kleinert

**Affiliations:** 1 Department of General, Visceral and Cancer Surgery University Hospital of Cologne Cologne Germany; 2 University of Cologne Cologne Germany; 3 Department of Haematology and Medical Oncology University Medical Centre Göttingen Göttingen Germany; 4 Department of Gastroenterology and Hepatology University Hospital of Cologne Cologne Germany; 5 Institute of Diagnostic and Interventional Radiology University Hospital of Cologne Cologne Germany; 6 Department II of Internal Medicine and Center for Molecular Medicine University of Cologne Cologne Germany; 7 Cologne Excellence Cluster on Cellular Stress Responses in Aging Associated Diseases University of Cologne Cologne Germany; 8 Department of Cardiology and Pneumology University Medical Centre Göttingen Göttingen Germany; 9 Division of Medical Education Research and Curriculum Development University Medical Centre Göttingen Göttingen Germany

**Keywords:** serious game, surgical education, clinical reasoning, virtual emergency department, medical education

## Abstract

**Background:**

Serious games enable the simulation of daily working practices and constitute a potential tool for teaching both declarative and procedural knowledge. The availability of educational serious games offering a high-fidelity, three-dimensional environment in combination with profound medical background is limited, and most published studies have assessed student satisfaction rather than learning outcome as a function of game use.

**Objective:**

This study aimed to test the effect of a serious game simulating an emergency department (“EMERGE”) on students’ declarative and procedural knowledge, as well as their satisfaction with the serious game.

**Methods:**

This nonrandomized trial was performed at the Department of General, Visceral and Cancer Surgery at University Hospital Cologne, Germany. A total of 140 medical students in the clinical part of their training (5th to 12th semester) self-selected to participate in this experimental study. Declarative knowledge (measured with 20 multiple choice questions) and procedural knowledge (measured with written questions derived from an Objective Structured Clinical Examination station) were assessed before and after working with EMERGE. Students’ impression of the effectiveness and applicability of EMERGE were measured on a 6-point Likert scale.

**Results:**

A pretest-posttest comparison yielded a significant increase in declarative knowledge. The percentage of correct answers to multiple choice questions increased from before (mean 60.4, SD 16.6) to after (mean 76.0, SD 11.6) playing EMERGE (*P*<.001). The effect on declarative knowledge was larger in students in lower semesters than in students in higher semesters (*P*<.001). Additionally, students’ overall impression of EMERGE was positive.

**Conclusions:**

Students self-selecting to use a serious game in addition to formal teaching gain declarative and procedural knowledge.

## Introduction

E-learning programs are now widely used for teaching declarative or theoretical knowledge [1,2]. One important part of clinical education is the transition of declarative knowledge to procedural knowledge [3], which is the basis for mastering clinical workflows in diagnosis and therapy or clinical reasoning. Virtual patient simulators [4] and serious games offer the possibility for teaching declarative and procedural [5] knowledge [6,7]. They are valuable in training specific medical scenarios that are hard to reproduce in the daily curriculum, such as rare clinical conditions, or are resource intensive (ie, major incident triage training) [8]. Even in common medical scenarios, serious games and virtual patient simulators enable virtual experience [9], as students can face the consequences of different decisions without putting real patients at risk.

Repetitive training allows the internalization of clinical patterns that are relevant for the necessary procedural performance [10]. Home-based distance learning with individual pace and number of repetitions allows equalization of students’ knowledge enabling effective face-to-face-teaching. Serious games extend the possibilities of virtual patient simulators because they are known to motivate students [11] as identification with an avatar (immersion) influences important incentives for intrinsic motivation, such as a sense of autonomy and a sense of achievement [12]. However, the amount of immersion depends on technical and, in particular, graphics quality, which should be “state of the art” [13,14]. The availability of serious games for clinical education is limited [15]. Commercial development of such a high-fidelity project is resource intensive [16], which may require subsidies because development outlay would necessitate high levels of sales to achieve profitability. In addition to technical quality, the success of serious games strongly depends on the amount of curricular content available [15]. Commercial serious games often lack medical content and are thus limited from a curricular perspective [17].

Therefore, it is desirable that the development of such educational methods is in the hands of universities because they are the main promoters of innovative educational methods and they are responsible for curricular content. However, the benefit of an educational tool of this kind is questionable if the impact on knowledge gain is still under discussion [11,15]. Moreover, most serious games are stand-alone solutions that cover only small aspects of clinical education [17] and are often not available outside published studies.

EMERGE is a serious game that combines both factors: it fosters training of clinical reasoning with state-of-the-art graphics that are likely to facilitate high immersion. We recently demonstrated the noninferiority of EMERGE compared to small-group teaching with respect to student learning outcome on clinical reasoning [18]. The aim of this study is to test EMERGE as an educational tool and determine the effect on student motivation and knowledge gain.

## Methods

### Description of the Serious Game

EMERGE allows free navigation through a virtual emergency department. A digital mentor supports the student while dealing with the interface and treating patients. When starting the simulation, students get information about the incoming patient from an emergency physician, they dispatch the patient to an examination room, take the patient’s medical history, order diagnostic tests, and establish a diagnosis and treatment. When taking the patient’s medical history, students are free to choose from an alphabetical list of 70 questions. Students can ask the questions in any order. If one of the questions is repeated, the virtual patient always responds with the same answer. Students are free to choose any diagnostic test or treatment that a modern emergency department offers. Students are not restricted to medically indicated tests nor to the sequence for taking these tests. All medical decisions have real consequences. For instance, patients respond to treatments and medications have effects and side effects. EMERGE was developed at Göttingen Medical School in collaboration with the University Medical Centre Hamburg-Eppendorf ensuring the educational quality of the program. EMERGE was programmed by PatientZero Games GmbH, at a cost of approximately €200,000.

EMERGE displays the classic features of a modern computer game: free interaction between the player and the game, a certain challenge (ie, a critically ill patient), a game story as the student plays a virtual doctor, and high immersion owing to high-fidelity graphics and an intuitive graphical user interface. A demo of EMERGE is freely accessible [17,19].

### Participants

Students of all clinical years (5th to 12th semester) of medical education at Cologne University (Cologne, Germany) were invited to sign up for the study via mailing lists, flyers, and social networks. Each semester consists of approximately 200 students; thus, 1600 students were invited. A total of 140 medical students (46 males, 94 females; mean age 24.1 years, age range 20-33 years; response rate 140/1600, 8.8%) volunteered to participate (see Table 1 for demographic information). Overall 67.1% (94/140) of our participants were female medical students. This distribution of male and female medical students is in line with the national average in Germany with approximately two-thirds of medical students being female [20]. Students received €15 for their participation. The Ethics Committee of the Medical Faculty at the University of Cologne approved the evaluation. The Institutional Review Board was informed, and there were no objections.

**Table 1:** Demographic information about the students who played EMERGE (N=140).

Variables		Male	Female
Number of students, n		46	94
Age, mean (SD)		24.5 (2.6)	23.8 (2.7)
**Semester, n**			
	5th semester	5	11
	6th semester	10	28
	7th semester	7	18
	8th semester	8	19
	9th semester	3	8
	10th semester	1	4
	Practical year (11th and 12th semester)	12	6

### Study Design

The effect of EMERGE on declarative and procedural knowledge was analyzed in a pretest-posttest design. Students first completed the questions measuring procedural and declarative knowledge. After students had played EMERGE, they again completed the questions measuring procedural and declarative knowledge. Additionally, after students had finished playing EMERGE, they also answered several questions measuring their impressions of EMERGE.

### Procedure

**Figure 1:**
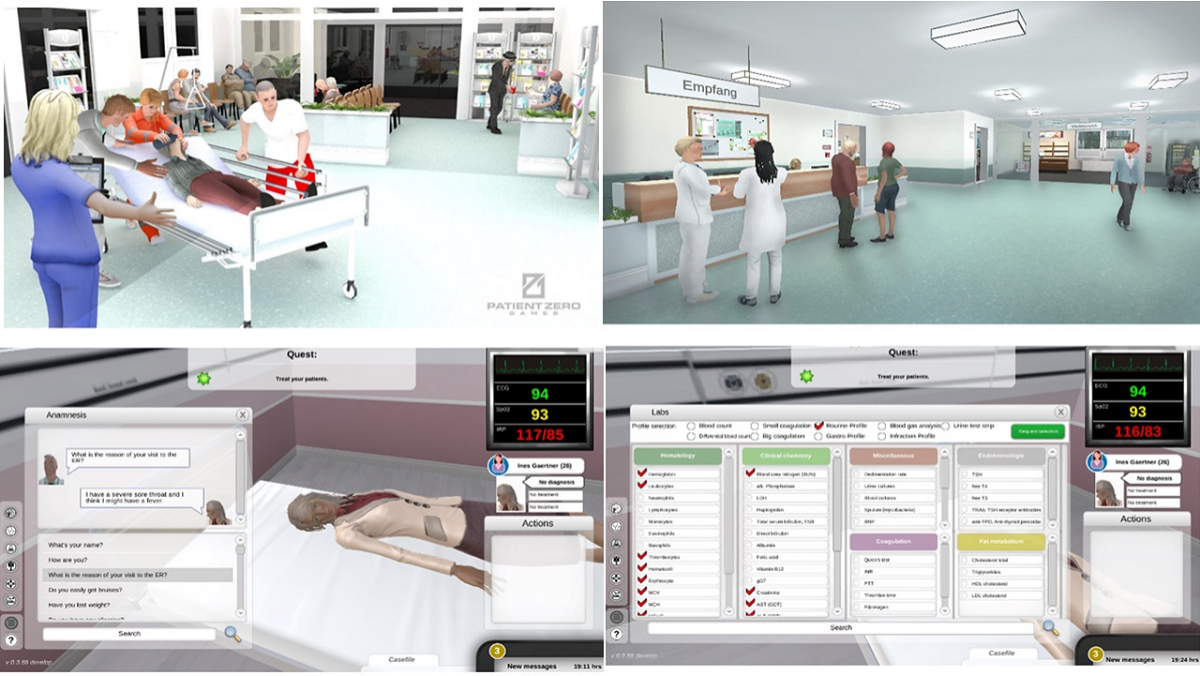
Sample screenshots of EMERGE. Students could freely interact with the environment.

In December 2017 and January 2018, 35 gaming sessions lasting 90 minutes each were conducted with four students allocated to each session (only four computers were available for this study). At the beginning of the experiment, students were greeted by the experimenter (FT). Each computer corresponded with one of four different case scenarios. Students were unaware of the associations between computers and case scenarios. Students were free to choose any of the four computers. After all students had taken their seats, they were instructed by the experimenter to complete the 20 multiple choice questions and the written Objective Structured Clinical Examination (OSCE) questions. Students were given 40 minutes to complete the questions. After that, students were instructed to imagine being a first-year intern in a real emergency department. Students were further instructed to behave as if their decisions had real consequences. After the instructions, the experimenter launched EMERGE and introduced them to the general controls of EMERGE. At the beginning of the game, students started their session in the entrance hall of the emergency department. A virtual map showed students the way to one of the various rooms within the department (Figure 1), where two virtual patients were already waiting for them. Students were free to decide which patient to interact with first. The time to play EMERGE was not limited. Students played for a mean 31.7 (SD 7.4) minutes. The game ended after students had diagnosed and treated the patients or transferred them to another department for treatment. At the end of the game, students were debriefed by a virtual doctor, who informed them about the correct diagnosis and treatment for each patient. After the students had finished playing EMERGE, they again completed the 20 multiple choice questions and the written OSCE questions.

### Medical Content

To increase the generalizability of the study and to ensure that the results would not be limited to just one condition, we created four distinct clinical cases: pneumothorax, sigmoid diverticulitis, mesenteric ischemia (easy), and mesenteric ischemia (difficult). These cases were selected because they are part of the standard surgical curriculum for German medical students [21]. At the University of Cologne, the surgical curriculum is spread out over several semesters. In the second clinical semester, students attend the main surgical lecture, covering all the declarative knowledge for the diseases used in this study. Additionally, students complete the first part of surgical bedside teaching. In the fifth clinical semester, students complete the second part of surgical bedside teaching. Moreover, as a part of the curriculum at the University of Cologne, clinical subjects are taught throughout the whole 6 years (12 semesters) of medical school, which results in students being presented with similar aspects several times during their training. For all diseases, correct diagnostic and treatment patterns were defined in process charts, as described in a previous article [22]. Briefly summarized, these process charts served as a blueprint for optimal diagnostic and therapeutic workflows. Congruence between student pathways and blueprints formed the basis for the assessment of student performance. Blueprints were based on published guidelines for each disease [23-25]. The four different clinical cases were combined into four different case scenarios (see Textbox 1). In each case scenario, students were presented with two medical conditions. Because the diagnosis of mesenteric ischemia requires profound clinical reasoning and experience, this condition was divided into an easy and a difficult case. In the easy case, serum lactate was high, and the patient suffered from an arrhythmia. These signs are strong indicators of this clinical condition. However, there is no laboratory value that is associated with mesenteric ischemia. A functional liver is able to eliminate lactate, even when there is a severe ischemic condition. Hence, the second case represented a patient without indirect signs, such as elevated lactate and arrhythmia. Due to restraints in the recruiting of participants for the study, only four different case scenarios could be tested. The cases of pneumothorax, sigmoid diverticulitis, and mesenteric ischemia (easy) were each used twice in the study. The case of mesenteric ischemia (difficult) was only used once. Because only 140 students participated in the study, it was not possible to create a fifth case scenario with an additional 35 participants to repeat the mesenteric ischemia (difficult) case.

**Combinations of the different clinical cases in the four groups (N=140).**

- Case scenario 1 (n=35)
  - Pneumothorax
  - Sigmoid diverticulitis
- Case scenario 2 (n=35)
  - Mesenteric ischemia (easy)
  - Sigmoid diverticulitis
- Case scenario 3 (n=35)
  - Pneumothorax
  - Mesenteric ischemia (easy)
- Case scenario 4 (n=35)
  - Mesenteric ischemia (easy)
  - Mesenteric ischemia (difficult)

### Impact on Declarative Knowledge

Students gain in declarative knowledge was determined by asking 20 multiple choice questions before and immediately after working with EMERGE. Influence of preexisting knowledge (ie, concordance validity) was measured by subgroup analysis with regard to study year.

### Impact on Procedural Knowledge

Students’ gain in procedural knowledge was measured by presenting students with a modified clinical case from OSCE describing a patient with sigmoid diverticulitis. This disease was intentionally selected for this analysis. Based on our own personal experience as OSCE examiners at our university, this case was the most complex of all the vignettes. Because the study lasted several weeks, it was expected that participants would exchange knowledge and experience. In order not to bias participants toward sigmoid diverticulitis, only half of the students playing EMERGE were presented with a patient with sigmoid diverticulitis in the game. In this subgroup, we were able to measure the effect of treating a patient with sigmoid diverticulitis on procedural knowledge. Time to read the case was not limited. After students read the case, they were asked the following six questions:

1. Please list the five most likely potential diagnoses for the patient.
2. What additional anamnestic questions would you ask the patient?
3. What procedures would you order to confirm your diagnosis and in what order?
4. How would you treat perforated sigmoid diverticulitis?
5. How would you treat nonperforated sigmoid diverticulitis?
6. How would you treat appendicitis?

Students had to write down their answers. Answers were compared to a blueprint created by two expert physicians (RK and SC) based on current medical guidelines and scored accordingly. Answers were scored by FT. Critical cases were discussed among all three authors (RK, SC, and FT) and resolved. Interrater reliability was not calculated.

**Figure 2:**
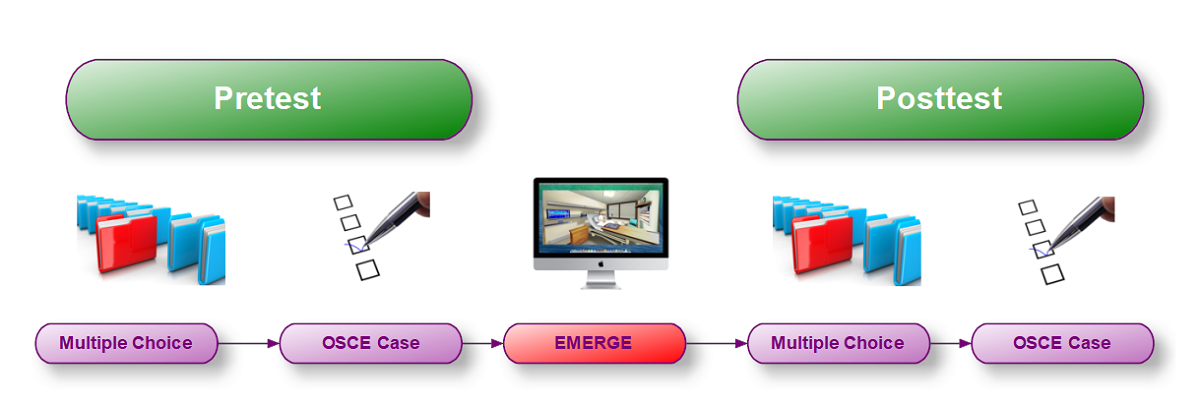
Testing declarative and procedural knowledge before and after working with EMERGE.

An overview of the experimental set-up can be seen in Figure 2. Pretest and posttest multiple choice questions and OSCE case were identical before and after EMERGE.

### Students’ Impressions of EMERGE

Students also rated 10 statements to measure three different aspects of their experience using EMERGE: (1) overall impression of EMERGE, (2) usability of EMERGE, and (3) student attitudes toward e-learning and computers. These 10 items had been used in a previous study on a Web-based immersive patient simulator [22]. Students rated these aspects on a 6-point Likert scale ranging from 1 (fully agree) to 6 (fully disagree). The overall impression of EMERGE was measured using the following statements: “Using EMERGE is fun,” “EMERGE teaches new knowledge,” “EMERGE prepares me for clinical practice,” “I would use EMERGE,” and “My overall impression of EMERGE.”

The usability of EMERGE was measured using the following statements: “EMERGE is easy to learn” and “EMERGE is easy to use.”

Student attitudes toward e-learning and computers were measured using the following statements: “I use computers on a daily basis,” “Computers, consoles, and cell phones are my hobby,” and “I mostly learn with books” (reversed item).

### Data Analysis

To assess students’ gain in declarative knowledge, students were presented with 10 multiple choice questions per clinical case. Because each student was presented with two clinical cases, students answered a total of 20 multiple choice questions. Students completed the same 20 questions before and after playing EMERGE. For each student, we calculated the percentage of questions they answered correctly. The percentage of correctly answered questions was analyzed in a mixed ANOVA. Effect sizes for ANOVAs are given as partial eta squared (η^2^). To determine the sample size for the study, a statistical power analysis was performed. This statistical power analysis was not based on data from prior studies but on general considerations about the trade-offs between the ability to detect certain effects and the feasibility to acquire a sufficiently large sample. Given a specified effect size (Cohen *d*), power, and alpha, the sample size was calculated using GPower (GPower 3.1). Because the implementation of new teaching methods can be expensive and time-consuming, potential new teaching methods should have a sufficiently large benefit. However, promising new teaching methods should not be overlooked. As a compromise, our study needed to be sufficiently powered to detect medium-sized effects with a Cohen *d* of 0.3 for the within-group comparisons. With an alpha=.05 and power=.80, the projected sample size needed to detect an effect of Cohen *d* of 0.3 for the within-group comparisons was N=90 (GPower 3.1). With an alpha=.05 and power=.80, the projected sample size needed to detect a large effect (Cohen *d* of 0.5) for the between-group comparisons was N=64 per group (GPower 3.1).

To assess students’ gain in procedural knowledge, we presented students with a clinical vignette (sigmoid diverticulitis) and asked them six questions about the vignette. Students provided their answers in written form. Because only half the students playing EMERGE were presented with a patient with sigmoid diverticulitis in the game, we were able to measure the effect of treating a patient with sigmoid diverticulitis on procedural knowledge.

Students’ answers to the first question were graded as correct if they had listed the correct diagnosis among the top three diagnoses. The gain in procedural knowledge was assessed by comparing the number of students who listed the correct diagnosis among the top three diagnoses *before* playing EMERGE to the number of students who listed the correct diagnosis among the top three diagnoses *after* playing EMERGE. The number of students before and after playing EMERGE were compared using generalized estimating equations (GEEs).

In the second question the number of diagnostic questions asked before and after playing EMERGE was analyzed using a mixed ANOVA.

Students’ answers to the third question were graded as correct if they had listed all diagnostic procedures in the correct order. Correct answers before and after playing EMERGE were compared using GEEs.

Students’ answers to the fourth, fifth, and sixth questions were graded as correct if they provided the correct treatment for each condition. Correct answers before and after playing EMERGE were compared using GEEs.

For all correlational analyses, Kendall tau (τ) was used as a robust measure of correlation. Data were analyzed using SPSS version 25 (IBM Corp, Armonk, NY, USA).

## Results

### Knowledge Gain: Impact on Declarative Knowledge

Working with EMERGE showed a positive impact on declarative knowledge. The percentage of correct answers to multiple choice questions increased from before (mean 60.4, SD 16.6) to after (mean 76.0, SD 11.6) playing EMERGE (*t*_139_=–13.92, *P*<.001, *d*=1.25).

**Figure 3:**
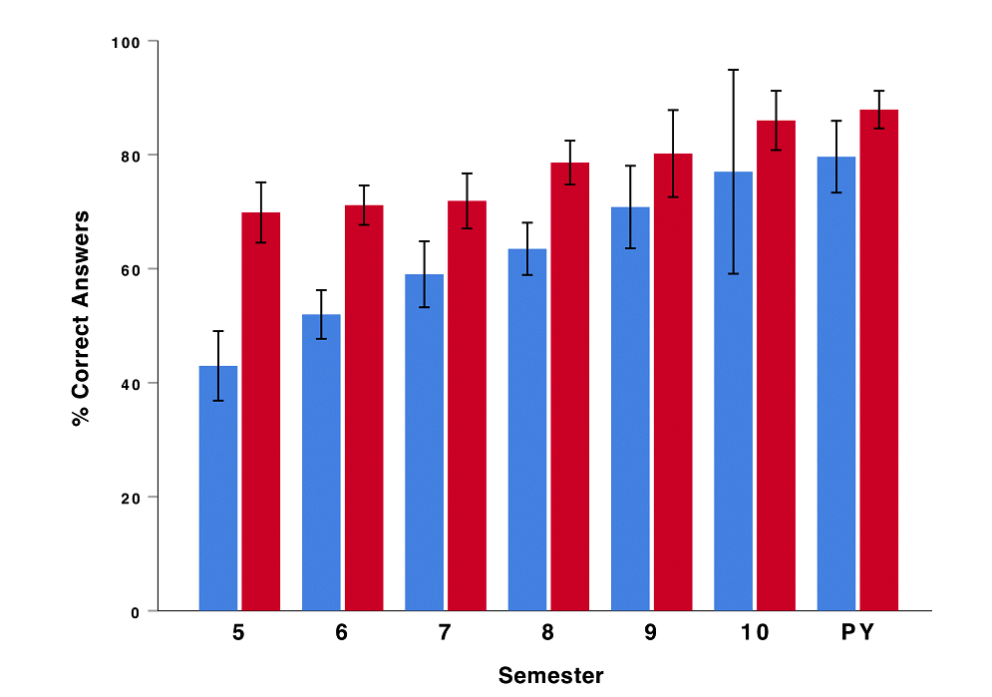
Percentage of correct answers by semester before (blue) and after (red) playing EMERGE. Error bars represent 95% confidence interval of the mean. PY: practical year (11th and 12th semester).

To test whether declarative knowledge differed between students in lower or higher semesters, we conducted a 2×7 mixed ANOVA (time×semester) as an exploratory analysis. There was a significant main effect of time on the percentage of correct answers (*F*_1,133_=130.67, *P*<.001, partial η^2^=.496). This suggests that participants answered significantly more questions correctly after playing EMERGE compared to before playing. There was also a significant main effect of semester (*F*_6,133_=4.76, *P*<.001, partial η^2^=.177), which suggests that students in higher semesters answered significantly more questions correctly than students in lower semesters. There was also a significant interaction effect between time and semester (*F*_6,133_=18.36, *P*<.001, partial η^2^=.453), which suggests that the knowledge gain after playing EMERGE was larger for students in lower semesters (see Figure 3). This was further supported by a negative significant correlation between knowledge gain (percentage of correct answers after playing EMERGE minus percentage of correct answers before playing EMERGE) and semester (Kendall τ_140_=–0.298, *P*<.001).

### Knowledge Gain: Impact on Procedural Knowledge

Students’ gain in diagnostic accuracy was analyzed using GEEs in a 2×2 design (time×sigmoid case). There were no significant effects (all Wald χ^2^_1_ <1.4). For descriptive results, see Table 2.

To test whether there was a gain in procedural knowledge, we compared the number of diagnostic questions asked in the sigmoid diverticulitis case before and after playing EMERGE in a 2×2 mixed ANOVA (time×sigmoid case). There was a significant main effect for time (*F*_1,138_=20.20, *P*<.001, partial η^2^=.128). All other effects were not significant (all *F*<.775). This suggests that students asked significantly more diagnostic questions after playing EMERGE (mean 3.11, SD 1.65) compared to before (mean 2.42, SD 1.38), regardless of whether they had been presented with a sigmoid diverticulitis case in the game or not.

To test whether the order of correct diagnostic procedures improved after playing EMERGE, we conducted an analysis using GEEs in a 2×2 design (time×sigmoid case). There was a significant main effect of time (beta=–2.54, Wald χ^2^_1_ =13.5, *P*<.001). All other effects were not significant (all Wald χ^2^_1_<2.8). This suggests that more students chose the correct diagnostic pathway after playing EMERGE (33/140, 23.6%) compared to before playing EMERGE (7/140, 5.0%), regardless of whether they had been presented with a sigmoid diverticulitis case in the game or not.

To test whether the treatment suggestions for the perforated sigmoid diverticulitis case improved after playing EMERGE, we conducted an analysis using GEEs in a 2×2 design (time×sigmoid case). There were no significant main or interaction effects (all Wald χ^2^_1_<0.9). For descriptive results, see Table 2.

To test whether the treatment suggestions for the nonperforated sigmoid diverticulitis case improved after playing EMERGE, we conducted an analysis using GEEs in a 2×2 design (time×sigmoid case). There was a significant effect of time (beta=–2.88, Wald χ^2^_1_=21.8 *P*<.001). There was also a significant effect of sigmoid case (beta=–2.46, Wald χ^2^_1_=14.7, *P*<.001). There was also a significant interaction effect (beta=2.51, Wald χ^2^_1_=14.5, *P*<.001). To break down this interaction effect, chi-square tests were performed. A significantly larger proportion of students who had been presented with a sigmoid case in EMERGE, 67 of 70 participants (96%), provided correct treatment suggestions after playing EMERGE than students who had not been presented with a sigmoid case, 46 of 70 participants (66%; χ^2^_1_=20.2, *P*<.001). There was no significant difference between both groups before playing EMERGE (χ^2^_1_=0.0, *P*=.87).

**Table 2:** Descriptive results of Objective Structured Clinical Examination before and after playing EMERGE for students presented and not presented a sigmoid diverticulitis case while playing EMERGE.

Questions	Before playing EMERGE, n (%)		After playing EMERGE, n (%)	
	No sigmoid case (n=70)	Sigmoid case (n=70)	No sigmoid case (n=70)	Sigmoid case (n=70)
Correct diagnosis	61 (87)	65 (93)	62 (89)	66 (94)
Correct diagnostic procedures	5 (7)	2 (3)	14 (20)	19 (27)
Correct treatment of perforated sigmoid diverticulitis	66 (94)	67 (96)	70 (100)	69 (99)
Correct treatment of nonperforated sigmoid diverticulitis	40 (57)	39 (56)	46 (66)	67 (96)
Correct treatment of appendicitis	67 (96)	66 (94)	68 (97)	67 (96)

**Table 3:** Mean ratings of the experience of using EMERGE (1=fully agree to 6=fully disagree).

Items	Mean (SD)
Using EMERGE is fun	1.68 (0.84)
EMERGE teaches new knowledge	1.98 (0.96)
EMERGE prepares me for clinical practice	1.95 (0.98)
I would use EMERGE regularly	2.22 (1.18)
My overall impression of EMERGE	1.91 (0.69)
EMERGE is easy to learn	1.61 (0.89)
EMERGE is easy to use	1.64 (0.91)
I use computers on a daily basis	1.63 (1.25)
Computers, consoles, and cell phones are my hobby	2.86 (1.52)
I mostly learn with books	3.49 (1.32)

**Table 4:** Correlations (Kendall tau) between semester and mean ratings of EMERGE.

Variables	Overall impression of EMERGE		Usability of EMERGE		Attitudes toward e-learning	
	Tau	*P* value	Tau	*P* value	Tau	*P* value
Semester	0.195	.002	0.058	.40	–0.091	.16
Overall impression of EMERGE			0.218	.001	0.122	.05
Usability of EMERGE					0.122	.07

To test whether the treatment suggestions for the appendicitis case improved after playing EMERGE, we conducted an analysis using GEEs in a 2×2 design (time×sigmoid case). There were no significant main or interaction effects (all Wald χ^2^_1_<0.2), which suggests that there was no difference in the proportion of students who listed the correct treatment for appendicitis before (133/140, 95.0%) and after (135/140, 96.4%) playing EMERGE.

### Impression Ratings of EMERGE

Students rated their overall impression of EMERGE (Cronbach alpha=.805; mean 1.95, SD 0.71) the usability of EMERGE (Cronbach alpha=.851; mean 1.62, SD 0.84), and their attitude toward e-learning (Cronbach alpha=.518; mean 2.66, SD 0.98). Table 3 shows the mean ratings of the students’ impressions of EMERGE. As Table 4 shows, there was a significant correlation between semester and overall impression of EMERGE, indicating that students in lower semesters had a more positive impression of EMERGE than students in higher semesters. There was also a significant correlation between the overall impression of EMERGE and the perceived usability of EMERGE.

## Discussion

The main goal of this study was to test EMERGE as an educational tool and to determine its effect on student motivation and knowledge gain. A pretest-posttest comparison yielded a significant increase in declarative knowledge. An exploratory analysis showed that this increase in declarative knowledge was smaller in more advanced students. With regard to procedural knowledge, a significant effect was noted for one of six categories, and this effect was only present in students who had actually been exposed to the respective case while playing the game. Only students that worked with the perforated sigmoid diverticulitis case also showed an increase in performance in the OSCE questions about nonperforated sigmoid diverticulitis, which is a sign for positive concordance validity [26].

There are several potential shortcomings that limit the generalizability of the results. For instance, the results may be influenced by the fact that participation in this study was on a voluntary basis and included mainly motivated students. Because motivated students are more likely to participate in experiments [27], future studies should investigate if less motivated students could also benefit from playing EMERGE. One way to study EMERGE’s effects on both motivated and less motivated students would be to integrate EMERGE into the regular curriculum.

Another limitation of our study is the fact that we did not study any long-term effects. Because our study focused only on a single intervention, future studies are needed to investigate if the effects of playing EMERGE can also be found after a longer period of time.

Also, we cannot disentangle the effects of EMERGE and other curricular activities because this was not a randomized controlled trial. It is important to note that the surgical curriculum is spread out over several semesters. Therefore, it is possible that some of the participants may have been exposed to some of the clinical conditions included in this study. However, because we did find a positive effect of playing EMERGE on knowledge gain, prior exposure was not strong enough to cancel out all potential learning effects.

For this study, case scenarios were limited to three diseases. Although students playing EMERGE did show an increase in declarative and—to a lesser extent—procedural knowledge, this was only true for the four clinical vignettes included in the study and procedural knowledge was only assessed in relation to one condition (ie, sigmoid diverticulitis). Thus, future studies should include a wider range of clinical cases.

However, it is important to identify the effect that leads to the knowledge gain. First, because this study lacks a control group, it is not clear what part of the knowledge gain can be attributed to playing EMERGE and what part is due to testing effects (ie, a gain in test scores because of familiarity with the questions). Second, at the end of the game students were debriefed by a virtual doctor who informed them about the correct diagnosis, so we cannot untangle the effect of playing EMERGE and the debriefing on knowledge gain.

Overall, students’ impression of EMERGE was positive. However, students in lower semesters had a more positive impression of EMERGE than students in higher semesters. Future studies should include more detailed measures of students’ impressions to further unentangle which groups of students are most likely to benefit from using EMERGE.

Beneath technical feasibility, curricular content of serious games plays a crucial role. Prior research on the impact of serious games on knowledge gain has been mixed—especially with regard to different levels of pedagogical strategies [15]. Therefore, using a third-party game in the curriculum may be risky because combining established pedagogical strategies with those used in serious games may be challenging. Hence, clinical teachers must adapt the cases in the game to their own curriculum to get a positive effect on knowledge gain.

Several prior studies have also shown positive effects of training with virtual patient simulators. For instance, Haubruck et al [28] showed that students who trained in chest tube insertions with the app Touch Surgery performed better than students who trained an unrelated skill. Additionally, Kowalewski et al [29] showed that virtual patient simulators have face and content validity. Also, Schwarz et al [30] showed that students generally have positive attitudes toward virtual platforms that train adaptive algorithms.

EMERGE shares certain features with immersive patient simulators [21]. However, it offers a higher degree of immersion because it expands on immersive patient simulators by adding features found in modern computer games, such as a high-fidelity game environment and the challenge of treating several patients in a limited amount of time. This opens the possibility of teaching nontechnical skills that are important in high-risk environments, such as the emergency department [22], but are hard to develop in an academic setting.

Lastly, working with EMERGE was widely accepted among students and was rated to be highly enjoyable by the students, which is an important precursor for motivated learning. Although this finding is commonly reported when evaluating serious games [31], it is still an important finding because, in addition to considerations of validity, creating enjoyable and motivating teaching interventions may be a goal in and of itself.

In this nonrandomized trial, we found significant gains in declarative and procedural knowledge in students self-selecting to use a serious game. Future studies need to determine whether this gain is attributable to the game itself.

## Abbreviations

GEE: generalized estimating equation
OSCE: Objective Structured Clinical Examination
